# Observations on the Non-occurrence of Secondary Attacks of Dysentery

**Published:** 1853-10

**Authors:** Austin Flint


*Observations on Non-recurrence of Secondary attacks of Dysentery*, By Austin Flint, M.D.—In a Clinical Report on Dysentery, based on an analysis of forty-nine cases in the course of publication in the *Buffalo Medical Journal*, Prof. Flint has a section devoted to observations on the "Recurrence of the Disease" which we copy entire.

The subjects of this section are interesting, and so far as I know have not been investigated by means of recorded facts. What remote effects, if any, in the organism, follow an attack of dysentery? Does it beget a predisposition to any other disease? Does it impair the constitutional vigor? Is the patient afterwards liable to renewed attacks of the disease, or is the susceptibility to the action of the cause, or causes, lessened, or destroyed, so that the morbid processes having once taken place, there is greater security, if not entire exemption thereafter? These are questions interesting in themselves, and important in their pathological bearings. The facts in this collection of cases pertaining to these subjects are not numerous, but few as they are, they lead to the belief that they involve something more than coincidences: that they point to laws of the disease which do not appear, as yet, to have received attention. I will proceed to present the results developed by the analysis.

Of the thirty-eight cases ending in recovery, in sixteen the patients either continued under observation, or definite information was obtained respecting their subsequent health for periods varying from one year to thirteen years. To be more precise, with regard to these periods, the duration of the subsequent history in the cases respectively are as follows: thirteen years, one case; seven years, two cases; five years, two cases; four years, nine cases; two years, one case, and one year one case. In all, sixteen cases. Of these sixteen patients only one has died. This patient died with tuberculosis of the lungs, four years after recoveriug from dysentery. Of the remaining fifteen cases, in all the health has been good during the periods, respectively, during which the patients remained under observation, or within knowledge. I mean by this, that not one has suffered from any other important disease, and in nearly every instance there has been excellent health.

*Not one of these fifteen patients has experienced a second*

*attack of dysentery.* The result is rendered somewhat more striking by the fact that, exclusive of the case in which the date of the disease was but a year since, all the patients have been for several years past within the sphere of an epidemic influence, the disease, as already stated, having prevailed more or less, as an epidemic, in this city, in the autumnal months for the last four years. I can also call to mind several cases of dysentery occurring at least four years since, the histories of which were not taken, the patients, in the mean time, being under observation. In none of these instances has the disease been twice experienced. *I have not yet met with a second attack of dysentery.* It is worthy of notice that three of the cases in this collection occurred in one family at different dates, viz., one case, thirteen years ago ; one four years and one two years: In these instances new subjects were attacked, they escaping who had previously had the disease.

It would seem, from these facts, that an attack of dysentery does not exert an unfavorable influenc on the organism, that patients are not rendered thereby prone to any particular disease, but, on the other hand, enjoy good health. It would also seem that patients are not liable to a repetition of the disease.

I would not be understood as presenting these inferences in the light of established truths. The number of observations is too small to warrant deductions which shall represent fixed laws of the disease. But, as already remarked, it is difficult to account for the facts on the hypothesis of their being due to mere coincidence ; and I repeat that the facts point to the existence of laws which remain to be settled by further investigation. We are at least justified in concluding, from the data before us, that deterioration of the health subsequent to dysentery is not the general rule, and that an attack does not lead to any increased susceptibility to the cause or causes of the disease.—*New-York Journal of Medicine.*

